# Postoperative Complications in implanted patients in the Cochlear Implant Program of Rio Grande do Norte - Brazil

**DOI:** 10.1590/S1808-86942010000400017

**Published:** 2015-10-19

**Authors:** Luiz Rodolpho Penna Lima, Fábio de Alencar Rodrigues, Clara Maria Dias Ferreira Calhau, Ana Carolina Dias Ferreira Calhau, Clara Terra de Paiva Palhano

**Affiliations:** a ENT specialized in Audiology and Cochlear Implant. PhD student, Coordinator of the Cochlear Implant Program of Rio Grande do Norte; b Fourth-Year Medical Student - Universidade Potguar; c ENT - Assistant Physician - Cochlear Implant Program - Rio Grande do Norte; d Third Year Medical Student - Universidade Potguar; e Second-Year Medical Student - Universidade Potguar

**Keywords:** cochlear implants, postoperative complications, surgery

## Abstract

Cochlear implant surgery is regarded as safe for the auditory rehabilitation of individuals suffering from profound/severe hearing loss. Complications may arise from the surgery. The complications of implant cochlear surgery reflect the operation complexity, the skill of the surgical team and the inherent risks of the procedure itself.

**Aim:**

To establish and discuss the postoperative complications in implanted patients from the Cochlear Implant Program of Rio Grande do Norte - Brazil.

**Study design:**

Retrospective analysis.

**Materials and methods:**

This paper discusses the clinical records of 250 patients implanted between August 2000 to December 2008. All patients were implanted by the same surgeon. The postoperative complications were classified in minor as those that resolved with minimal or no treatment and major as those requiring additional surgery or hospitalization.

**Results:**

In our sample, 33 patients (13.2%) had postoperative complications. Minor complications affected 20 cases (8.0%), while major complications occurred in 13 cases (5.2%). Hematomas, device failures and infections had the highest clinical relevance.

**Conclusion:**

This review reinstates the safety of the surgical procedure in relation to the possible occurrence of postoperative complications and emphasizes the need for continuous surgeon education and training.

## INTRODUCTION

Cochlear implants (CI) are electronic devices which enable the auditory rehabilitation of individuals with severe to profound bilateral hearing loss^1^, aiming at electrically stimulating the auditory nerve fibers^2^, in such a way as to replace cochlear function. Throughout the world over 120,000 patients have been implanted with different types of devices, and results are improving on a daily basis.

The Cochlear Implant Program (CIP) of Rio Grande do Norte has implanted 250 pediatric and adult individuals from August of 2000 through December of 2008, made up of a multidisciplinary team of physicians, speech and hearing therapists, psychologists and social workers. This experience has enabled a compilation of statistical data concerning the postoperative complications found in this service compared to the cochlear implant programs throughout Brazil and in the world.

The postoperative complications of the surgical procedure represent some of the most frustrating and difficult to deal by surgeons, and involve different variables intrinsically associated with the patient and the surgical team. Such complications reflect the procedure's complexity, surgeon's skill and risks inherent to the deep insertion of a large foreign body below the scalp^3^.

The surgery has been greatly changed, especially during the last fifteen years, aiming at reducing the incidence of medical per and postoperative complications, besides the efforts of the device manufacturing companies to correct system failures. Despite all of this, complications may happen, having an incidence of 12% in Cochlear Implant Centers in the USA^4^.

Surgical complications may be classified into major - if they require additional surgery or hospitalization, and in minor, when they resolve in an outpatient ward or even with no treatment at all, as advocated by Cohen et al. (1988). Major complications involve meningitis, flap necrosis, device failure, electrode extrusion, facial nerve paralysis and others; while the minor complications involve facial nerve stimulation, electrode migration, vertigo, tinnitus, and others. The major complications which require surgery review and, especially those associated with device insertion are not common^5^.

In a study carried out in Latin America involving 40 cochlear implant centers in 10 countries, with a sample of 3,768 implanted patients, there were 193 postoperative complications (5.1%), and the major causes were: spontaneous system failure (86 cases), skin inflammation caused by the magnet (35 cases) and infection (26 cases). In this study, the author concluded that such complications are commonly associated to the type of cochlear implant^6^.

American Cochlear Implant Centers reported an incidence of major and minor complications of 8% and 4.3%, respectively, in a sample of 2,751 implanted patients^3^.

In another study, such centers revealed complication rates of 5% and 7%, respectively in a smaller series of 459 implanted patients^4^.

Postoperative complications in a sample of 100 implanted patients in the specialized center of Birmingham, UK were reported as major in 3.2% of the cases^7^. A similar study was carried out in Izmir, Turkey; in a pediatric sample with 227 implantees, the incidence of major and minor complications were 12.33% and 6.6%, respectively^8^.

Relatively high incidences have been observed in Manchester from June of 1988 to June of 2002 in a sample of 240 adult implantees, in whom they observed 6.25% and 25.4% of major and minor complications, respectively^9^. In an experience limited to 30 implantees in Italy, there were two major complications, corresponding to 6.6% of the cases^10^.

In Brazil, a similar study was carried out in São Paulo, with a series of 35 implantees, and they concluded that the cochlear implant surgical complications are directly associated to the surgeon's skill^11^. The variation in implant surgery complication incidences and the few Brazilian studies on these regards corroborate the need to carry out such a study in the Cochlear Implant Center in Rio Grande do Norte with the aim of finding the statistical data in a significant sample.

## METHODS

Our cohort study included 250 charts from adult and pediatric patients submitted to cochlear implant surgery at the Cochlear Implant Center of Rio Grande do Norte, from August of 2000 to December of 2008. As far as hearing loss etiology goes, 40.1% was idiopathic. Some 21.5% of the patients had congenital rubella as cause; 6.8% corresponded to meningitis sequelae and 6.8% of the patients had it because of ototoxic agents; 24.8% of the patients had other etiologies. All the procedures were carried out by the same surgical team.

The clinical reports analyzed included patient data such as age and gender, preoperative evaluation, intraoperative and postoperative results. The data was collected from the medical charts of implanted patients from the Cochlear Implant Program information database.

The patients were previously explained about the study and research upon admission to the Cochlear Implant Program through a free and informed consent form. The study was submitted to ethical evaluation and was approved under CAAE protocol # 0232.0.052.000-08.

The variables were compiled and the graphs were made in Microsoft Excel.® The statistical data was submitted to analysis through the EPIINFO 6.0® software.

For didactic reasons, the major complications were defined as those requiring another surgery, implant removal and those which caused significant medical problems. The minor complications were defined as those which were spontaneously resolved or treated at an outpatient basis with minimum conservative treatment.

## RESULTS

There were complications in 33 cases (13.2%). The mean age of patients who had complications was 16.26 years and the median age was 4.35 years. Of these, 45.4% and 54.6% were males and females, respectively, proving that gender was not a risk factor for complications in our series. Complications happened to 24 children (72.7%) younger than 7 years, 23 of them were younger than 5 years; 6 were adults (18.2%) and 3 were elderly (9.1%) -older than 60 years. We stress that none of these patients had anatomical malformations.

As to the cochlear implant model used in the surgery of the patients who had some type of complication, 57.5% were Nucleus Contour and 21.2% were Nucleus 24 K. In 12.1% of the cases, the model used was Medel C 40+ and in 9.2% of the cases we used the Medel Pulsar.

**Graph 1:**
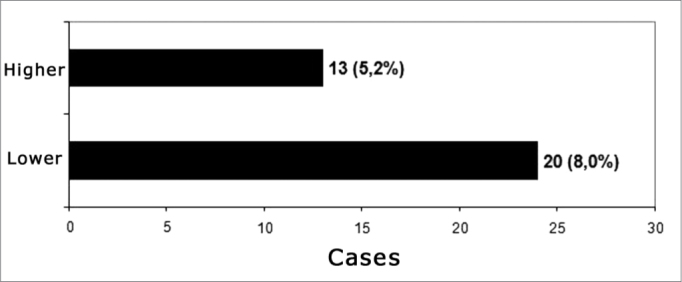
Postoperative complications classification according to Cohen & Hoffman (1988)

Minor and major complications were seen in 20 cases (8.0%) and 13 cases (5.2%), respectively - Graph 1. A similar incidence was seen in the USA Implant Centers^3^; in a sample of 2,751 patients, there were 339 complications (12.3%).

### Minor Complications

The minor complications were treated conventionally. Vertigo was seen in 6 cases, nonetheless, the symptoms did not continue in any of them. Moreover, of the six cases, one patient had more than one minor complication - vertigo and tinnitus.

There were 5 cases of surgical flap infection. They ware all treated with conventional antibiotics. There were 5 cases of suture dehiscence, of which 4 happened because a certain suture wire was used during the procedure and one case happened by inadequate handling by the patient. After conventional treatment, healing happened by second intention.

One patient had hematoma which regressed after the first week of postoperative without surgical intervention. There was one case in particular in which we there was pneumomediastinum and tinnitus. The list of minor complications can be seen on Table 1.

**Table 1:** Minor complications in implanted patients in the Rio Grande do Norte CIP (n = 250).

Minor complications	Number of cases	% of the total
Vertigo	6	2.4
Infection	5	2.0
Suture dehiscence	5	2.0
Tinnitus	2	0.8
Hematoma	1	0.4
Pneumomediastinum	1	0.4
TOTAL	20	8.0

### Major Complications

Table 2 summarizes the major complications found in the sample of patients implanted by the Rio Grande do Norte Cochlear Implant Program. The incidence was not influenced by the hearing loss etiology or by patient gender.

**Table 2:** Major complications in implantees of the Rio Grande do Norte CIP (n = 250).

Major complication	Number of cases	% of the total
Hematoma	4	1.6
Device failure	3	1.2
Infection	3	1.2
Ground electrode shifting	1	0.4
Flap thickening	1	0.4
Gain reduction	1	0.4
TOTAL	13	5.2

The reimplant surgery can be indicated for many reasons, including device failure, extrusion or substitution of the internal unit for another device^12^. In most of the cases the procedure is technically safe and the audiologic results are satisfactory^5^. Of the thirteen patients who had major complications, the reimplant surgery was done in 4; and from these, 2 were because of device failure and 2 were because of infection. Surgery requires considerable attention to details. The skin over the implant is frequently atrophied and must be handled carefully. Moreover, the fibrous and bone tissues are in the surgical bed, making it difficult to recognize the anatomy.

In a review done with 28 reimplanted patients because of device failure, it was concluded that approximately a quarter of these patients had insufficient performance after the reimplant procedure^13^. Of the 4 patients who underwent reimplant surgery in our center, all had satisfactory responses.

## DISCUSSION

The importance of the surgeon having proper training and skills in order to do the cochlear implant surgery must not be underestimated. Attention to details concerning the surgical technique can help avoid numerous complications^3^^,^^5^, although those not associated with the surgery itself are significantly important and cannot be foreseen by the surgeon.

The companies which manufacture the cochlear implant devices must strive to minimize the incidences of device failure, since they produce considerable psychological trauma to family and patients alike, besides increasing morbidity. Although the incidence of device failure is low, it is advisable to consider preventive measures in order to avoid those secondary to trauma^5^, such as the use of a helmet in order to practice physical activities. As it happens in any surgical procedure, periodically there is the need to reassess the procedure by the cochlear implant centers in its entire context, from pre to postoperative, with the aim of developing protocols in order to reduce the risk of major and minor complications. The cochlear implant surgery has become increasingly safer thanks to a dynamic process in which implant centers and manufacturers work together.

The major complications happened to 5.2% of the cases. This incidence is similar to those reported in other studies of the same nature, which rates vary between 3 and 13.7%^7^^,^^14^, ^15^, ^16^. Of the 13 patients who suffered major complications, the reimplant procedure was carried out in 4 patients. Besides the psychological trauma to the patient, this brings about severe financial implications which must be taken into account when one calculates the cost of a cochlear implant program.

Of the 5 patients with postoperative hematoma, surgical drainage was done in 4, therefore being considered major. Of these, only one was diagnosed with coagulation disorder. They all had a satisfactory improvement after the procedure.

In the USA implant centers^3^, from a sample of 2,858 cases, the device failed in 52 implantees, corresponding to 1.8%. In Latin America, there have been 104 cases (2.76%) of spontaneous system failure or secondary to trauma in a sample of 3,768 patients, associating them to the type of implant, and the failures happened more commonly to the ceramic type of implant^5^. In our center, 4 patients had implant failure (1.6%). Among the 3 device failures, the reimplant procedure was carried out in 2 cases and one patient refused to be reoperated.

Although infection is uncommon, it represents a severe complication of the cochlear implant surgery^14^. In our sample, one patient had otomastoiditis on the thirtieth day of post-op because from a skin lesion infected by Varicela Zoster virus, and the patient was reimplanted. There was a second patient infected by S. Aureus, who was also reimplanted. In another case, there was a skin infection caused by Pediculus humanus capitis, whom had the abscess drained. These infections affected children in a mean age of three years, suggesting a greater susceptibility in this age range concerning the development of postoperative infections. Because of the smaller head side and the fact that children have a device identical to the one implanted in adults, we can foresee a higher number of complications in children^3^. Infection is a complication which still concerns the surgeon, having seen that progression with later implant removal is associated with greater patient morbidity and a higher cost to public coffers. Device infection is commonly associated with bacterial biofilms and seems to be invulnerable to an immune response^18^^,^^19^.

In one case the ground electrode shifted after device malfunctioning. The patient was submitted to reoperation in which we noticed implant intactness and its shifting. The ground electrode was repositioned, and there was a satisfactory improvement of implant functioning.

Numerous studies showed that the most frequent major and minor complications reported were associated with the surgical flap incision^3^^,^^11^. In only one case there was a flap thickening because of the impossibility of coupling the antenna to the device. After thinning out the flap, the device worked satisfactorily.

Recently, a high incidence of meningitis was reported in implanted patients^17^. We reinforce that in the Cochlear Implant Program of Rio Grande do Norte there were no cases of postoperative meningitis. Immunization against Streptococcus Pneumonnie and Neisseria Meningitides is part of the program protocol.

We stress the lack of deaths, internal unit migration, facial nerve paralysis/paresis, iatrogenic cholesteatoma, electrode bundle extrusion and seroma in patients implanted in the Rio Grande do Norte Cochlear Implant Program.

## CONCLUSION

And finally, we reinstate the safety of the surgical procedure as to the possible complications arising from the surgery. There were complications in 33 patients (13.2%). Major and minor complications corresponded to 5.2% and 13% of the cases, respectively. The incidence of major complications is low and most of the minor complications can respond satisfactorily to conservative treatment. The benefits greatly outweigh the risks, and it is worth stressing that the safety of the cochlear implant procedure is directly associated to the surgeon's continued education and training.
